# Assessing the nutritional information for children younger than two years old available on popular websites

**DOI:** 10.1016/j.rppede.2016.03.004

**Published:** 2016

**Authors:** Gisele da Silva Gomes Monteiro, Maíra Macário de Assis, Maria Alvim Leite, Larissa Loures Mendes

**Affiliations:** a Departamento de Nutrição, Instituto de Ciências Biológicas, Universidade Federal de Juiz de Fora, Juiz de Fora, MG, Brazil; b Departamento de Saúde Pública, Escola de Medicina, Universidade Federal de Juiz de Fora, Juiz de Fora, MG, Brazil; c Departamento de Nutrição, Escola de Enfermagem, Universidade Federal de Minas Gerais, Belo Horizonte, MG, Brazil

**Keywords:** Internet, Child nutrition, Food Guide

## Abstract

**Objective:**

To analyze whether the information found on popular Internet sites are in accordance with the steps recommended by the Food Guide for Children Younger than Two Years of the Ministry of Health (2010).

**Methods:**

Descriptive/comparative study, carried out between August and October 2014, which carried out a search for popular sites (for lay people) in Portuguese, containing information on nutrition of children younger than two years. The Google search engine was used. These findings were compared with the Food Guide for Children Younger than Two Years of the Ministry of Health (2010). It was verified whether the information shown on the websites was in accordance with the Guide.

**Results:**

A total of 50 sites were analyzed, including blogs, food company websites and websites specialized in child nutrition. Only 10% of those pages correctly showed every step of the Food Guide. The recommendations were: exclusive breastfeeding up to six months of life (80%); complementary feeding from six months of life (36%); baby food consistency according to the guide (48%); encouraging the consumption of fruits and vegetables daily (60%). Regarding the complementary feeding safety and hygiene, 26% contained correct information. Only 36% correctly warned about which foods should be avoided in the first years of life.

**Conclusions:**

The information found on the sites is largely in disagreement with the Ministry of Health recommendations, which can lead to misconceptions in the nutritional care of the children younger than two years.

## Resumo

**Objetivo:**

Analisar se as informações veiculadas em sites populares estão de acordo com os passos recomendados no Guia Alimentar para Crianças Menores de Dois Anos do Ministério da Saúde (2010).

**Métodos:**

Estudo descritivo/comparativo, executado entre agosto e outubro de 2014, no qual foi feita uma busca por sites populares (para leigos) na língua portuguesa que continham informações sobre a alimentação de crianças menores de dois anos. A ferramenta de busca usada foi o Google. As informações encontradas foram comparadas com o Guia Alimentar para Crianças Menores de Dois Anos do Ministério da Saúde (2010). Foi verificado nos sites se a informação apresentada estava de acordo com o Guia.

**Resultados:**

Foram analisados 50 sites, entre os quais blogs, sites de empresas alimentícias e sites especializados em nutrição infantil. Apenas 10% dessas páginas apresentaram corretamente todos os passos do Guia Alimentar. As recomendações foram: aleitamento materno exclusivo até o sexto mês (80%); alimentação complementar a partir dos 6 meses de vida (36%); consistência da papa salgada de acordo com o Guia (48%); incentivo ao consumo de frutas, verduras e legumes diariamente (60%). Sobre a segurança e a higiene da alimentação complementar, 26% continham informações corretas. Somente 36% alertavam corretamente sobre quais alimentos devem ser evitados nos primeiros anos de vida.

**Conclusões:**

As informações contidas nos sites apresentam, em grande parte, discordância com o que é preconizado pelo Ministério da Saúde, o que pode acarretar equívocos no cuidado nutricional com os menores de dois anos.

## Introduction

The early years of a child's life, especially the first two, are characterized by a rapid growth and great evolution in the development process. This is a vital period for the implementation of good eating practices, given that it is in this phase that the abilities to begin digestion of breast milk and other foods are acquired and important physiological changes regarding intake self-control take place, which may determine the food standard in adulthood.^1^^,^ 2

In Brazil, over ten million users access health websites regularly. A study conducted in 2011 revealed that most people who search the Internet for health information do so for their own health or that of their relatives. Women represent the majority of users.^3^ Currently, the Internet is used by three billion people worldwide, according to a study published in November 2014 by the International Telecommunication Union (ITU) and publicized in several countries by the United Nations (UN). The use of the Internet has been steadily growing worldwide; from 2013 to 2014, it increased by 6.6%. Such use has doubled from 2009 to 2014 in developing countries.^4^ In the Information and Communication Technologies Development Index (IDI), which ranks countries considering their level of access to these technologies and the use of Internet, Brazil has advanced from the 67th position in 2012 to the 65th in 2013, reaching a rating of 5.5. According to that document, in 2013, 51.6% of all Brazilians used the Internet; 48.8% of homes in the country had a computer, and of those, 42.4% had Internet access. It is estimated that 40 million new wireless broadband Internet connections were made in 2013.^4^ Regarding the profile of Internet users, most of them referred its use for learning and education purposes; the proportion of women who access the Internet for such information is greater than men.^5^

The Ministry of Health recommends exclusive breastfeeding up to 6 months; from that age onwards, the introduction of complementary feeding is indicated. In the exclusive breastfeeding period, offering teas, juices, and water is unnecessary, since breast milk is a complete food and the normal newborn is born with relatively high hydration levels.^6^ In addition, offering fluids in the bottle can harm the baby's sucking ability, reduce the intake of breast milk, and decrease their willingness to suckle again on the breast.^1^^,^ 6 ^,^ 7 From the sixth month onwards, although breast milk continues to feed and protect infants against diseases and is an important source of calories and nutrients, exclusive breastfeeding is no longer able to meet all their nutritional needs and energy; thus, the introduction of new foods is justified.^6^^,^ 7 The early introduction of low nutritional value and high-calorie foods in the first years of life and the reduction of exclusive breastfeeding are factors that contribute to the emergence of allergic processes, eating disorders, anemia, and overweight, in addition to decreasing the protective ability of the immune system.^2^^,^ 8

Despite the growing access of mothers, fathers, and caregivers to the Internet for information on breastfeeding and complementary feeding, resorting to popular websites that have no scientific content, there are still few Brazilian studies that address this theme. A search in the Scientific Electronic Library Online (SciELO) with the use of descriptors “Internet” and “complementary feeding” retrieved only one reference.^9^ Thus, the present study aimed to analyze whether the information on popular websites is in accordance with the steps recommended in the 2010 Food Guide for Children Under Two Years of the Brazilian Ministry of Health.^1^

## Method

This was a descriptive/comparative study, conducted during August 2014. The study considered the importance of promoting good eating habits in the age range of up to 2 years and the lack of autonomy on the part of the children regarding their choices. The focus was to analyze websites accessed by parents and caregivers. A search for popular websites in Portuguese aimed at the general public, which contained information about feeding children under 2 years of age, was conducted. The definition of the number of surveyed sites was made after a simulation of a circumstantial search made by a lay individual, encompassing all initial sites retrieved in the search that met the research purposes.

The search engine used was Google, due to its wide distribution and ease of use by the lay public. After this stage, the process of website selection and analysis began. The keywords used were “infant feeding,” “complementary feeding,” “baby feeding,” and “first baby food.” All websites that met the defined selection criteria and that were available on the Internet at the time of data collection were included in the study. Sites targeted toward healthcare professionals were not analyzed.

Data collection was conducted by a nutrition student through a checklist prepared with the Ten Steps to Healthy Eating, available in the Food Guide for Children Under Two Years of the Brazilian Ministry of Health,^1^ which are: (1) exclusive breastfeeding until the age of 6 months, with no water, tea or any other food; (2) at 6 completed months, slowly and gradually introduce other foods, maintaining breastfeeding up to the age 2 years or more; (3) at 6 completed months, give complementary foods (cereals, tubers, meat, legumes, fruits and vegetables) three times a day, if the child is still being breastfed; (4) complementary feeding should be offered according to the family meal times, at regular intervals, and respecting the child's appetite; (5) complementary feeding should be thick from the beginning and be offered with a spoon, starting with a pasty consistency (porridge/purees) and gradually increasing the consistency until reaching that of the family's meals; (6) children should be offered different foods throughout the day (a varied diet is colorful); (7) encourage the daily consumption of fruits and vegetables at meals; (8) avoid sugar, coffee, canned, fried foods, soft drinks, candies, and other treats in the early years of life; salt should be used sparingly; (9) pay attention to food hygiene, preparation, and handling; ensure adequate storage and conservation; (10) encourage the sick and convalescent child to eat by offering their favorite foods and respecting the child's acceptance.

Through a checklist, each of the information found on the websites that was related to the ten steps included in the Guide was compared and classified as follows: (I) in accordance with the Guide, (II) in violation of the Guide, or (III) did not have the information sought.

## Results

A total of 50 websites were assessed; all of them were classified as unscientific, including blogs, food companies’ websites, and websites specializing in child nutrition. Some were administered by healthcare professionals, mostly physicians and nutritionists. The summary of the results is shown in Table 1, in which it appears that only 10% (*n*=5) of analyzed sites correctly presented the ten steps presented in the Food Guide for Children Under Two Years, published by the Brazilian Ministry of Health in 2010.^1^ Regarding the first step of the Food Guide, which addresses complementary feeding initiation, only one site (2%) did not have this information. The majority, 82% (*n*=41), were compliant with the recommendation that breastfeeding should be exclusive until the age of 6 months, and that the introduction of water or teas is not required. However, eight websites (16%) presented the information that the exclusive breastfeeding period should be until the fourth month, when other liquids, such as natural juices, should be introduced.

**Table 1:** Information collected on popular websites, compared with the Food Guide for Children Under Two Years (Ministry of Health, 2010); Brazil, 2015.

Recommendation	*n*	%
*Step 1-Exclusive breastfeeding until the sixth month*		
Agreement	40	80
Disagreement	9	18
No information	1	2

*Step 2-Complementary feeding from the sixth month onwards*		
Agreement	18	36
Disagreement	12	24
No information	20	40

*Step 3-Food offer*		
Agreement	32	64
Disagreement	13	26
No information	5	10

*Step 4-Meal times according to that of the family*		
Agreement	15	30
Disagreement	5	10
No information	30	60

*Step 5-Consistency*		
Agreement	24	48
Disagreement	13	26
No information	13	26

*Step 6-Varied food*		
Agreement	33	66
Disagreement	2	4
No information	15	30

*Step 7-Daily offer of fruits and vegetables*		
Agreement	30	60
Disagreement	2	4
No information	18	36

*Step 8-Avoid offering processed foods*		
Agreement	18	36
Disagreement	2	4
No information	30	60

*Step 9-Importance of hygiene during preparation*		
Agreement	13	26
Disagreement	0	0
No information	37	74

*Step 10-Encourage the sick child to eat*		
Agreement	6	12
Disagreement	0	0
No information	44	88

Regarding the second step of the Guide, which recommends complementary feeding starting from the sixth month onwards, 36% (*n*=18) of the sites presented the correct information, while 24% (*n*=12) presented it incorrectly, and 40% (*n*=20) omitted it.

After analyzing the third step, it was found that 64% (*n*=32) of the websites properly informed that complementary foods should be offered after the sixth month, three times a day, for children who are exclusively breastfed. Another 26% (*n*=13) said only one daily baby food would be required to supplement the feeding at this stage, or that only fruit should only be offered, while the Guide recommends the introduction of cereals, tubers, meats, vegetables, fruits, and vegetables. The remaining 10% (*n*=5) did not feature this information.

On the fourth step, most of the websites (60%; *n*=30) did not mention this information, while only 30% (*n*=15) presented it correctly and 10% (*n*=5), incorrectly.

The fifth step indicates that complementary feeding should be initiated in the form of porridge and purees, and should be served with a spoon. It is necessary that the increase in consistency occurs gradually, until reaching that of the family meal. The result found on popular sites was that 26% (*n*=13) still indicated that the meal should be sifted or liquefied to prevent choking.

Regarding the sixth step, in the present study, most of them (66%; *n*=33) were faithful to the information contained in the Guide and 30% (*n*=15) did not have this guidance. Only 4% (*n*=2) of the sites presented incorrect information about this step, as they encouraged the supply of processed or sugary foods.

One of them, presented in the seventh step of the Food Guide, concerns the encouragement of daily consumption of fruits and vegetables at meals, for which 60% of the sites provided the correct information.

The eighth step indicates that the following foods should be avoided in the first years of life: sugar, coffee, canned food, fried foods, soft drinks, candy, snacks, and other treats; salt should be used sparingly. Although it is an extremely important step, only 36% (*n*=18) of the sites presented it correctly, 4% (*n*=2) presented it incorrectly, and most (60%; *n*=30) did not give this information.

The vast majority of sites (74%; *n*=37) did not feature information about complementary food safety and hygiene, while 26% (*n*=13) contained correct information, which corroborated the ninth step: attention to food hygiene, preparation, and handling; ensure proper storage and conservation.

Only 12% (*n*=6) of the websites addressed the tenth step, which is to encourage sick and convalescent children to eat by offering their usual food and favorite foods, respecting their acceptance. Most websites 88% (*n*=44) did not present this recommendation.

## Discussion

Regarding to step one, which reinforces the exclusive breastfeeding until six months of age, it is important to highlight that a study that assessed the growth of children receiving exclusive breastfeeding until 6 months demonstrated that these children reached the sixth month with a mean weight higher than the 50th percentile of the National Center for Health Statistics (NCHS) weight curve.^10^ This reinforces that breast milk not only ensures the development of numerous psychological and immunological skills, but also is a complete food from a nutritional standpoint and, when provided to the child exclusively until the age of 6 months, promotes healthy and proper growth at this stage, meeting all the nutritional needs and reducing the incidence of morbidity and mortality, among many other benefits.^10^^-^ 12

On the second step, exclusive breastfeeding is able to nourish the child only until the sixth month; after this age, it is necessary to introduce new foods. At this stage, the child is already physically and psychologically prepared to receive complementary foods. Early or late introduction of complementary foods can cause serious damage to the child's health and development. If started early, it reduces exclusive breastfeeding time and may decrease the absorption of important nutrients present in breast milk; if started late, it can lead to deficiency in micronutrients that are essential to child development.^7^^,^ 13

About third step, complementary food should be rich in micronutrients and energy, have a proper consistency, and be uncontaminated, to ensure a healthy growth of the child. Iron, for example, is an essential micronutrient for growth and infant development. The introduction of foods can interfere with the absorption of the iron present in breast milk, so it is very important that complementary foods are iron-rich, such as meat or offal, and are sources of vitamin C, which contributes to the absorption of non-heme iron present in foods such as dark green vegetables.^1^^,^ 7 ^,^ 13

The time when complementary feeding is introduced in the child's routine is very important for a better acceptance during this transition phase, as well as is addressed on the fourth step.^1^ This step clarifies that complementary foods should be served according to the family meal time, at regular intervals, and respecting the child's appetite. This is one of the steps that were modified in 2010, since the previous Food Guide, from 2002, stipulated that complementary feeding should be offered without rigid schedules, always respecting the child's will.

Regarding the fifth step, the consistency of complementary foods is very important, as a proper texture stimulates the chewing motion and provides enough calories to meet the child's need at this stage. Food must be mashed into a puree consistency, rather than diluted, sifted, or liquified, because the latter methods eliminate the need for chewing and the foods significantly lose their nutritional value, thus hindering oral dynamics, growth, and proper development.^1^^,^ 14 ^-^ 16

In a study that evaluated scientific and popular sites, Werneck^16^ found that the information present in the sixth step, which indicates that the child should be offered different foods each day, was correctly presented in 41.7% of the sites, while 58.3% did not provide any information about food diversity. Different from the present study, which had more encouraging results, and most evaluated websites (66%) were true to the Guide's recommendation (step 6).

Regarding the step 7, the acceptance of fruits and vegetables by children cause great anxiety in parents. The rejection of these foods is common, because in addition to the fact that food neofobia is a physiological characteristic, children usually have a preference for sweet taste. There are, however, several ways to establish healthy eating habits early.^16^^,^ 17

Despite the importance of the eighth step, which recommends avoiding the offer of some foods such as sugar, coffee, canned, fried foods, soft drinks, candies and other processed foods to children under two years, most of the web sites (60%) omitted this information. Such foods, besides reducing children's appetite, compete with nutritious food, making children reject the latter. The introduction of energy-dense foods with low nutritional value from the beginning of life and the early withdrawal of breastfeeding contribute to impaired growth and development, and lead to decreased immune protection, triggering of allergic processes, and nutritional disorders.^1^^,^ 8 ^,^ 10 ^,^ 18 ^,^ 19

About the ninth step, attention to hygiene in the preparation of meals is very important to prevent infections and food poisoning through contaminated food. In this stage of life, children are still developing their immune system and need greater attention and care. In prepared food, the proliferation of organisms can occur if the food is left at room temperature or if the fridge is not kept at a proper temperature (below 5°C). The recommendation is that food should be prepared in sufficient quantity for the time of consumption.^1^^,^ 6 ^,^ 7

On the tenth and final step, it is of utmost importance that the sick child is fed properly for a quick recovery, preventing delays in growth and weight loss, which at this stage can become irreversible, and reinforcing the role of parents and caregivers in the stimulus of feeding.^1^^,^ 20

It is worth emphasizing that this study has some limitations, such as the fact that social networks such as Facebook and Instagram, which have shown an increase use in the country in recent years, were not included. Furthermore, the quality of the websites, including specific criteria such as legibility, completeness, design, and accuracy, among others, was not broadly assessed.^21^ Nevertheless, in the context outlined in the present study, it is important to mention the role of healthcare professionals at all levels of attention in providing reliable and updated information, in line with the recommendations of official health agencies, to demystify potential misinformation disseminated on the Internet.

The information contained in the websites was, for the most part, in disagreement with what is recommended by the Brazilian Ministry of Health (2010). The information that was most in contradiction with the recommendation was that relating to complementary feeding, both in the diversity of the food supply and in its consistency. Still, it should be noted that a considerable number of the analyzed sites reported the importance of exclusive breastfeeding until the sixth month. It was observed that, although content on food and nutrition have gained space on the Internet due to the increased demand of the population for health-related topics, many sites present incorrect information, without theoretical foundation.

Therefore, greater publicity of the appropriate recommendations and the inspection of content on websites focused on child feeding are necessary. Reliable sources directed both to the lay population and healthcare professional can contribute to disease prevention and health promotion.
